# Supporting continuous glucose monitoring for people with serious mental illness and type 2 diabetes: a co-design study for a structured intervention

**DOI:** 10.3389/frhs.2026.1807689

**Published:** 2026-08-20

**Authors:** Jennifer V. E. Brown, Olivia Matthias, Jessica Hendon, Ramzi A. Ajjan, Najma Siddiqi, Ian Kellar, Peter A. Coventry

**Affiliations:** 1 Department of Health Sciences, Faculty of Sciences, University of York, York, United Kingdom; 2 School of Medicine and Population Health, Faculty of Health, University of Sheffield, Sheffield, United Kingdom; 3 Hendon Creative, York, United Kingdom; 4 Division of Cardiovascular and Diabetes Research, School of Medicine, University of Leeds, Leeds, United Kingdom; 5 Leeds Teaching Hospitals NHS Trust, Leeds, United Kingdom; 6 Hull York Medical School, York, United Kingdom; 7 Bradford District Care NHS Foundation Trust, Bradford, United Kingdom; 8 School of Psychology, Faculty of Science, University of Sheffield, Sheffield, United Kingdom; 9 School of Nursing and Public Health, Faculty of Health and Education, Manchester Metropolitan University, Manchester, United Kingdom

**Keywords:** co-design, continuous glucose monitoring, intervention development, serious mental illness, type 2 diabetes

## Abstract

**Introduction:**

Individuals with serious mental illness (SMI), such as schizophrenia or bipolar disorder, are at a two- to threefold increased risk of developing type 2 diabetes and face significant health inequalities, including reduced life expectancy. Diabetes self-management in this population is challenging, and existing interventions are poorly suited to their needs. Continuous glucose monitoring (CGM) offers potential benefits but remains underused. This study aimed to co-design a logic model and programme theory for a structured CGM intervention tailored to adults with SMI and type 2 diabetes, as a resource to support the planned future co-production of the content and the implementation of the full intervention.

**Methods:**

Using experience-based co-design, this study engaged service users, carers, and healthcare professionals (HCPs) in workshops and discussions to identify challenges and facilitators for CGM use. Participants co-developed an outline of candidate intervention components, which were summarised in a logic model and programme theory. The process included synthesising existing evidence, exploring personas, and mapping CGM user journeys. The draft intervention outline was iteratively refined across multiple sessions.

**Results:**

Ten participants (four HCPs, four service users, and two carers) contributed to the co-design process. Barriers identified included limited technical support, accessibility challenges, and a lack of integrated physical and mental healthcare. Key components for a structured CGM intervention include personalised CGM training, flexible delivery methods, ongoing support, and peer networks. The proposed intervention was conceptualised in three phases: set-up, early days, and living with CGM. Participants prioritised flexibility and tailored intervention adjustments, emphasising the importance of addressing both physical and mental health needs concurrently.

**Discussion:**

This study demonstrates the feasibility of co-designing interventions with individuals often considered “hard to reach.” Our study highlights that people with SMI can take an interest in their physical health and see the potential for CGM to improve diabetes management, provided they receive appropriate and flexible support. Future work will develop and refine the intervention and evaluate its clinical and cost-effectiveness in diverse populations, aiming to address significant unmet needs in diabetes care for this vulnerable group.

**Study registration:**

https://osf.io/4ae3g/ (2 February 2024).

## Introduction

### Serious mental illness and type 2 diabetes

Individuals with serious mental illness (SMI), such as schizophrenia, bipolar disorder, and psychosis, are two to three times more likely to develop type 2 diabetes than the general population (1–3). They also face an increased risk of diabetes-related complications and poorer health outcomes (4). The coexistence of diabetes and other chronic conditions contributes to a reduced life expectancy of 15–20 years (5). This “mortality gap” is internationally recognised, yet interventions to address it have been largely ineffective (6). Despite extensive research and policy efforts, individuals with SMI continue to experience poorer health and higher mortality rates, primarily due to physical health issues.

Furthermore, people with SMI are often systematically excluded from health research due to restrictive eligibility criteria or inaccessible methods, which limits their access to healthcare innovations and reinforces profound inequalities (7–10). They engage with healthcare services differently and frequently require additional support to access care effectively (11, 12). Consequently, they may not fully benefit from existing interventions that fail to address their specific needs (13, 14). While self-management interventions show moderate effectiveness for mental health (15), diabetes self-management remains underdeveloped and particularly challenging in this population (16, 17). This challenge is compounded by underlying psychological factors; research demonstrates that psychological vulnerability in patients with diabetes is strongly linked to behavioural inhibition systems and anxiety sensitivity, underscoring the need to address mental health concurrently with physical health management (18). Moreover, existing educational programmes, such as DESMOND, do not adequately address the complexities of managing both diabetes and SMI (19–21).

### Advances in diabetes care through continuous glucose monitoring

The use of technology in diabetes care is expanding globally, with continuous glucose monitoring (CGM) transforming care delivery and patient experience. Its power lies in its potential to drive behaviour change, a capacity that remains largely untapped, particularly for people with SMI. Structured glucose monitoring—which integrates the technology with support, education, and feedback—enhances diabetes management, lowers HbA1c, and improves quality of life (22). Such support is especially crucial for individuals with SMI, who often struggle to access quality healthcare without additional assistance (23). For individuals with SMI, who may face challenges with aspects of diabetes self-management that require memory, attention, and decision processes (24), CGM is particularly promising. Its ability to provide immediate, visual feedback on how food and activity affect glucose levels can act as a powerful behavioural prompt (25). This real-time visual reinforcement can help build self-efficacy and support lifestyle changes in ways that traditional blood glucose monitoring fails to provide, given the sporadic and limited nature of the data (26).

Even though CGM's availability continues to expand, individuals with SMI receive little consideration (27, 28), despite its potential to improve diabetes self-care (29). In the United Kingdom, HbA1c is measured annually in this population during routine health checks (30), but access to specialised diabetes care remains limited, perpetuating health disparities (31). To begin addressing these gaps and to harness the potential of new technologies for this population, targeted research is essential to explore how CGM can enhance diabetes self-management, improve glucose control, and promote overall health in people with SMI.

### Including people with SMI in CGM research

We have begun investigating the feasibility and acceptability of CGM for people with SMI (32). To avoid adverse events from overwhelming amounts of data, we asked participants to wear a blinded sensor, i.e., one without a companion app or reader. We found high uptake and good data completeness, supporting the notion that CGM is feasible in this population. Qualitative interviews revealed that participants had a positive experience and recognised that unblinded CGM (i.e., with a companion app) could be beneficial for their diabetes self-management through increased diabetes awareness and the sensor acting as a behavioural prompt. Participants wanted a summary of their glucose data, which we provided after the study; they needed support in interpreting these data summaries. It was also apparent that participants needed support with device fitting and removal.

The present study builds on these initial findings by adopting a person-centred co-design approach. The goal was to develop a logic model and programme theory for a structured CGM intervention for people with SMI.

### Theoretical foundations and guiding frameworks

The behaviour change wheel (BCW) (33), a synthesis of 19 theoretical frameworks, serves as the overarching strategic model for this study. We draw on the principles of systematic behavioural diagnosis using the COM-B model (which stipulates that Capability, Opportunity, and Motivation determine Behaviour) and its granular expansion, the theoretical domains framework (TDF) (34), to identify barriers and facilitators to engagement with CGM among people with SMI. We will map these insights to behaviour change techniques (BCTs) (35), which are active intervention ingredients. By identifying the corresponding mechanisms of action (MoAs) (36), we provide a further breakdown of how the new intervention is hypothesised to affect change.

This co-design study was conducted within the context of existing interventions, including CGM and various diabetes education and self-management programmes. It combined evidence on CGM and diabetes care with research on the healthcare needs of people with SMI, integrating insights from service users, carers, and healthcare professionals (HCPs). The aim was to develop a logic model and programme theory for a structured intervention that enables individuals with SMI to incorporate CGM into their diabetes management. The study followed the Medical Research Council's (MRC) guidance on complex intervention development, which highlights the importance of programme theory—clarifying how an intervention is expected to work—to enhance methodological transparency and rigour (37). Stakeholder involvement in intervention design (38) improves acceptability, and this study aimed to (1) collaboratively define the key components of the new structured intervention and (2) systematically conceptualise these within a logic model and programme theory for refinement and testing. Rather than adapting an existing intervention, this represents the creation of a novel approach (39).

This study has been reported in accordance with the GUIDED (GUIDance for the rEporting of intervention Development) framework (40), with a completed checklist provided in Supplementary Appendix 1.

### Target population, study aim, and objectives

The new intervention is targeted at adults (aged 18 and over) living in the community who have confirmed diagnoses of both type 2 diabetes and SMI (schizophrenia, schizoaffective disorder, psychosis, bipolar disorder). Specifically, we focused on the following objectives:

1. To explore the potential benefits and challenges of CGM for people with SMI and type 2 diabetes
  (a) With service users and carers
  (b) With HCPs
2. To identify candidate components for a structured CGM intervention for people with SMI and type 2 diabetes with service users, carers, and HCPs
3. To prioritise candidate components for a structured CGM intervention for people with SMI and type 2 diabetes with service users, carers, and HCPs
4. To synthesise outputs from objectives 1–3 to develop a logic model and programme theory

## Methods

**Figure 1:**
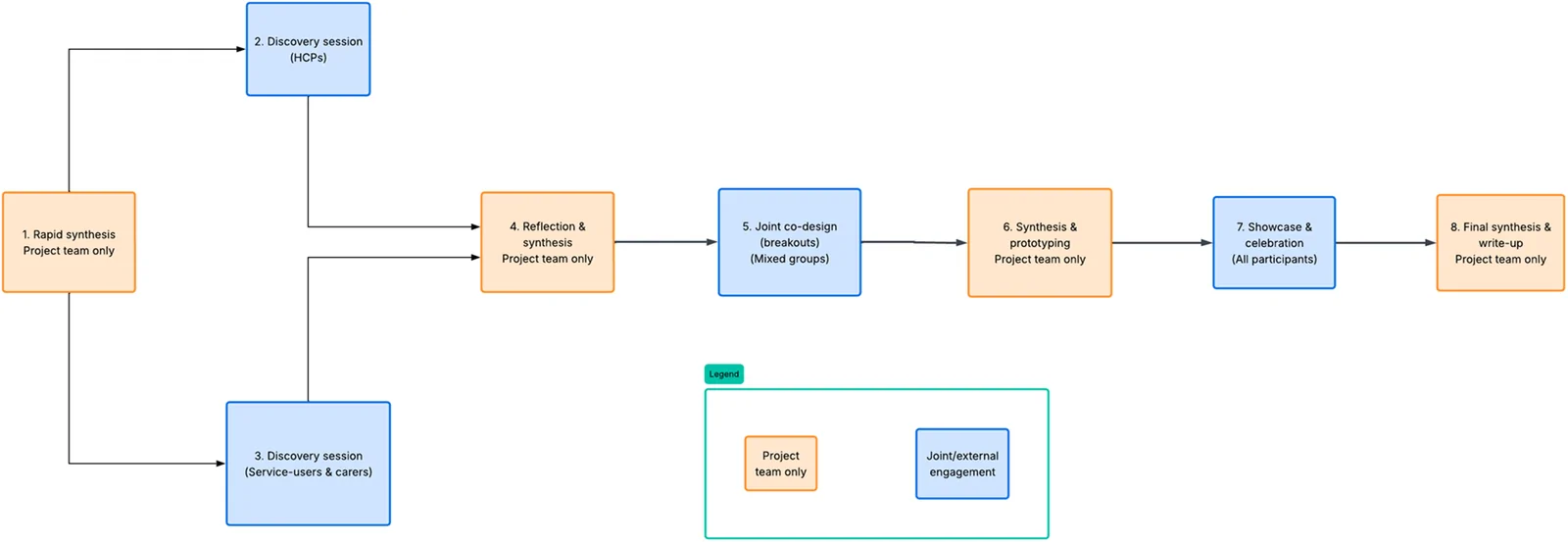
Project steps.

We conducted an accelerated co-design study of potential intervention components, using experience-based co-design (EBCD) methods (41) alongside other knowledge translation strategies known to facilitate co-design, such as persona development, animations, and prioritisation tasks (42). We used existing evidence as well as theory to accelerate the first phase of the project and presented catalyst materials in simplified form to stimulate rapid idea generation. The project steps are summarised in Figure 1 and described as follows.

### Project team

The project was led by JB, a behaviour change researcher with a background in health psychology and extensive experience in applied health research. JB was supported by PC, RA, IK, and NS, senior researchers with expertise in health services research, diabetes, health psychology, and psychiatry, respectively. The project also benefited from the involvement of an experienced independent co-design facilitator, JH (43). In addition, a student nurse (adult nursing) on a research internship (OM) supported the project.

### Participants

Recruitment took place between 1 June and 31 August 2024. Our goal was to recruit five service users, five carers, and five HCPs (total *N* = 15). We recruited service users and carers from existing participant cohorts who had consented to be contacted about future research opportunities. In addition, we asked participating service users to identify informal carers who might be interested in taking part. We followed the King's Fund definition (44) of informal carers, which is endorsed by the NHS and the Department of Health and Social Care, and includes anyone caring for “a relative or friend who needs support because of… illness, including mental illness,” by providing “active support, supervision, or social interaction.” HCPs were recruited through existing networks and contacts. Participants gave written informed consent at the beginning of the study. At the start of each workshop, they confirmed their ongoing consent verbally, with a hand signal, or by giving a “thumbs up” on a video call. Supplementary Appendix 2 provides a detailed description of the recruitment and consent process, along with examples of participant-facing documents.

We offered service user and carer participants £25/h (i.e., £75 per 3-h session). We did not offer payment to HCP participants for their time. We paid travel and associated expenses for all participants.

#### Eligibility criteria

Table 1 summarises the eligibility criteria for the three groups of participants described above.

**Table 1:** Eligibility criteria for participation.

All participants		
Criterion	Inclusion	Exclusion
Age	18 years or older	Under 18 years
Capacity	Capacity to consent	No capacity to consent
Cognitive impairment	No or mild cognitive impairments no affecting ability to provide informed consent	Any cognitive impairment that affects the ability to provide informed consent
Language^a^	Ability to communicate in spoken English	No or severely limited ability to communicate in spoken English, e.g., requiring the use of an interpreter
Time commitment^b^	Commitment to attending and contributing to at least two out of three sessions (discovery session, co-design workshop, celebration event)	Not able to commit to at least two out of three sessions

Additional criteria for service users		
Criterion	Inclusion	Exclusion
SMI diagnosis	At least one of the following Schizophrenia Schizoaffective disorder Bipolar disorder Psychosis Severe depression Patients with or without other co-occurring mental health conditions are eligible	Any other mental health diagnosis, regardless of severity or duration No mental health diagnosis
Diabetes diagnosis	Type 2 diabetes (insulin-treated or non-insulin-treated) Patients with or without other co-occurring physical health conditions are eligible	Any other type of diabetes, including Gestational diabetes Type 1 diabetes Diabetes secondary to other health conditions, e.g., genetic defects
Living situation	Community dwelling Living in supported housing or residential settings with some degree of autonomy over day-to-day activities	Current inpatients

Additional criteria for carers		
Criterion	Inclusion	Exclusion
Caring responsibilities	Current or recent unpaid carer of a person meeting the criteria outlined above for service user participants, e.g., a family member, spouse, or friend Carers are eligible to take part regardless of whether the person they care for (service user) is taking part in the study	Paid, i.e., professional, carers Family members, spouses, or friends of service users who do not provide care

Additional criteria for HCPs		
Criterion	Inclusion	Exclusion
HCP role and experience	Any HCP, regardless of job role or seniority, with current or recent experience in at least one of the following: Primary care, e.g., annual physical health checks for people with SMI Mental healthcare delivered in community-based secondary care settings for people with SMI, e.g., clozapine clinics, community mental health teams, early intervention psychosis clinics Diabetes care delivered in secondary care settings, e.g., diabetes clinics	Any other HCP, including Mental health professionals without direct experience working with people with SMI Mental health professional working exclusively with inpatients HCPs from any specialty not relevant to mental health or diabetes

a *Language*: We did not formally assess prospective participants' level of English, but a sufficient grasp of the language was crucial for participation in this study. Where possible, we made adjustments, for example, by reading aloud study information materials and limiting the amount of reading and writing participants were asked to do.

b *Time commitment*: Participants had the right to stop their participation at any point, without having to provide a reason. However, at the point of recruitment, prospective participants were informed that participation involved attending at three events, and they were encouraged to commit to attending at least two out of the three.

### Ethics statement

This study was approved by the Department of Health Sciences Research Governance Committee (HSRGC) at the University of York, York, UK, on 9 April 2024 (reference number HSRGC/2024/616/G).

### Project steps and materials

The project steps are outlined in the study protocol (see https://osf.io/4ae3g/). Supplementary Appendix 3 describes changes from the protocol. Throughout the study, the project team (JB and IK) and facilitator (JH) took detailed notes to record group discussions. Examples of the workshop materials are available in Supplementary Appendix 4.

#### Step 1: rapid synthesis of existing evidence and preparation for discovery sessions

To stimulate rich conversations and rapid idea generation, we summarised existing evidence to create a catalyst slide deck containing the following information:

- An introduction to the health inequalities faced by people with SMI, including their elevated risk for diabetes and poorer outcomes;
- An overview of CGM systems and their role in diabetes care;
- Information that people with SMI type 2 diabetes are currently not eligible for NHS-funded CGM, unless they meet specific additional criteria;
- Links to resources about the FreeStyle Libre (Abbott) system available in the United Kingdom;
- A high-level summary of findings from our mixed-methods systematic review about the acceptability of CGM across the entire population with diabetes (publication pending), highlighting that overall acceptability is good but that support with the interpretation of CGM data to drive behaviour change is considered key; and
- A summary of previous findings (32), drawing out high levels of CGM uptake, participant requests to receive data summaries, and high levels of engagement using illustrative quotes from people with SMI and type 2 diabetes.

**Figure 2:**
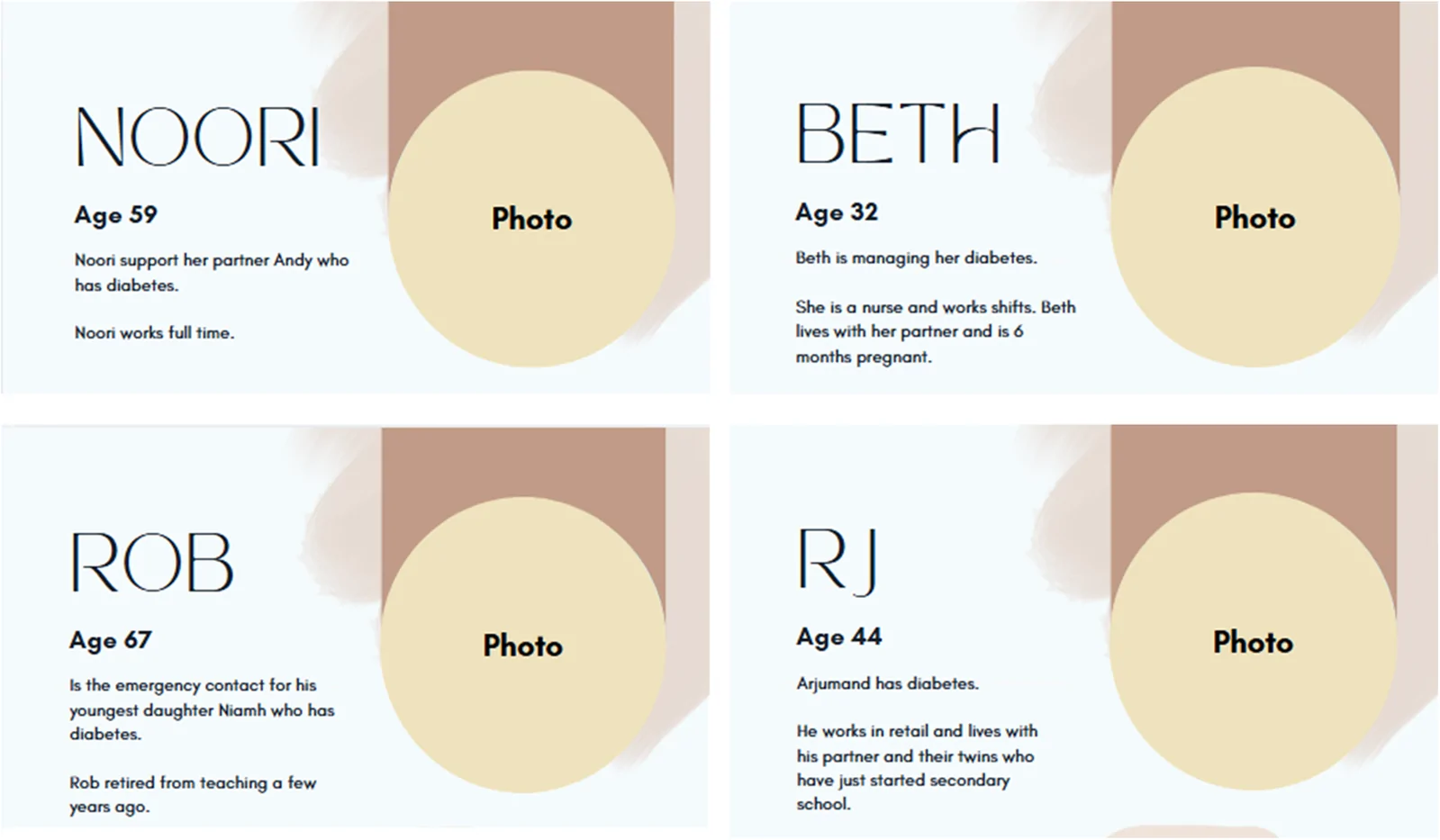
Persona sketches prepared for the discovery sessions.

We created two service user personas and two service user/carer dyads. At this stage, minimal information was provided (name, age, diabetes status, employment status), as illustrated in Figure 2. We chose to use fictional personas rather than real-case vignettes to provide a safe and creative participatory method for service users and carers. This approach encourages candid feedback and allows participants to project their needs and ideas without disclosing their own, potentially sensitive, personal experiences (45).

In parallel, we developed sets of persona prompts to use during the discovery sessions to “flesh out” the personas and develop insights into their experiences with SMI and diabetes. Box 1 below summarises the prompts we used during the session.

Box 1 Session prompts.

- What is the health status of [persona]?
- Are there any fact's about [persona's] life you'd like to note?
- What about [persona] (and their family) in terms of their day-to-day outlook?
- How are they getting on in the workplace or at school?
- How are family relations going?
- How is diabetes affecting all of this? Give some examples.
- Does [persona] have a CGM? How are they using it and does it affect anything you have noted above?

#### Steps 2 and 3: discovery sessions

Discovery sessions for HCPs (group 1, *N* = 4) and service users and carers [group 2, *N* = 3 (1 carer + 2 service users)] were held on Zoom (in line with participant preference). Following the presentation of the catalyst materials, participants were introduced to the persona sketches. Participants worked in small groups to develop the personas, creating fictional backstories for their lives. Participants were encouraged to consider the impact of diabetes and SMI on the personas' outlook and daily routines.

Small-group work was interspersed with whole-group segments, during which personas were introduced to the other participants and their circumstances were described in some detail. In the final part of the discovery sessions, participants were asked to consider CGM in the context of the personas' lives and circumstances.

#### Step 4: reflection, synthesis, and preparation for joint co-design session

**Figure 3:**
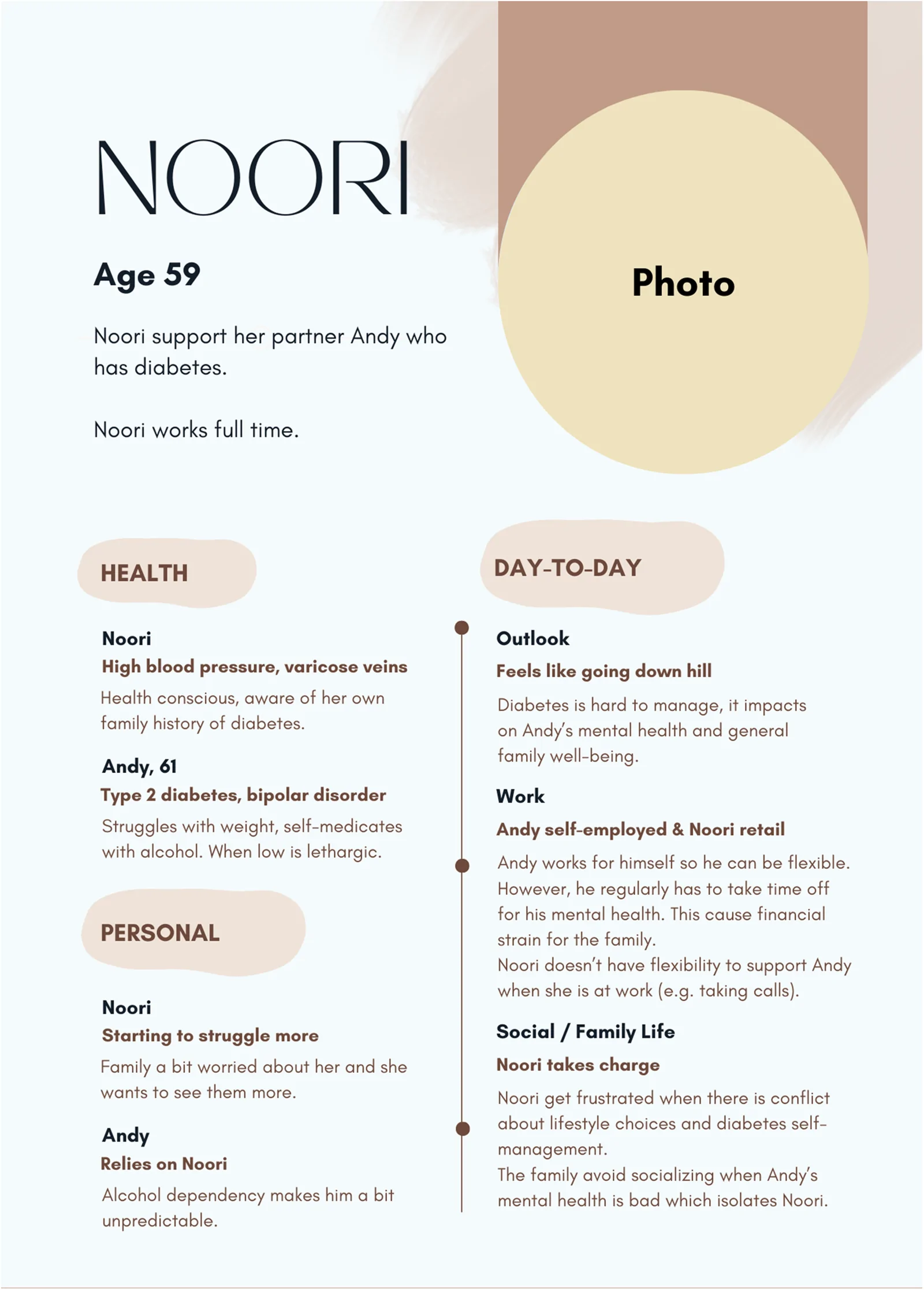
Summary persona sketch for Noori.

After the discovery sessions, we integrated HCPs', service users', and carers' ideas to create one version of each persona. Animations were created for two personas to introduce variety in the delivery of the next session and to increase the accessibility of the persona-based approach by “bringing to life” the fictional characters. Figure 3 shows the summarised persona sketch for Noori, a fictional carer for her partner Andy, who has bipolar disorder and type 2 diabetes.

During the synthesis work, it became apparent that the opportunities and challenges that had been created in the discovery sessions for each of the personas could be grouped into three distinct but overlapping phases of the CGM journey: (1) set-up, (2) early days, and (3) ongoing maintenance. We qualitatively synthesised the barriers and facilitators reported across the different stages by categorising our workshop notes to identify descriptive themes, which are presented in the Results section.

Following a journey mapping approach, we prepared CGM journey sheets with prompts to stimulate discussion and support the identification of candidate components. Journey mapping in healthcare is a method used to visualise and analyse a patient's experience as they navigate through different stages of the healthcare system, to identify areas for improvement in service delivery and patient care (46).

#### Step 5: joint co-design event with breakout groups

For this session, all participants (service users, carers, and HCPs) worked with the project team. The event was held in a hybrid format to maximise participants' opportunity to attend. All members of the project team were present in the meeting room with the in-person participants. One member of the project team provided specific facilitation for the online participants.

Following an introduction to the synthesised and fully developed personas, participants were asked to consider each stage of the CGM journey (set-up, early days, ongoing maintenance). The group was split up so that one breakout group focused on the carer perspective and the other focused on the service user perspective. Each breakout group was facilitated by the project team, who guided the conversation and took notes. Initially, participants were asked to follow the journey stages of for each persona. However, where discussions deviated from this outline, we allowed the conversation to flow freely, provided relevant content was still being produced. Box 2 below shows the completed journey sheet for Noori with notes taken during the session.

Box 2 Completed journey sheet.

Noori

- Andy's being offered a CGM.
- Noori has some worries at the back of her mind.
- Andy is hoping the family will help with the tech side of things.

Set-up

**Table:** 

What happens during set-up?	Problems?	What helps solve the problem?
Missed first appointment due to admin error, then missed second appointment due to alcohol, could not drive, and Noori was at work	SMI is not in the criteria, so there is a need to make people aware of it, as it might impact the process	Easy to fall through the cracks during the letter phase if you have an SMI, so the system needs to acknowledge that
This has delayed the whole process, as it bounced back to the GP due to a missed referral. GP only picked up missed appointments at a regular checkup	Could it be up to 6–18 months delay on when to get CGM, due to delays in getting to appointments, etc.?	SMI flag on the system. Supported engagement rather than just letters
Physical health deteriorated in the meantime, and the link to missed appointments was not made (e.g., giving frequent prescription of antibiotics should be treated as a red flag, urine infections led to missed opportunities to intervene)	Various missed appointments for physical health	Use of text messages
		Ability to fast-track
		GP oversight to ensure it goes through to the first appointment
		Diabetes in the community: easier access to services
		Open clinic for that first set-up rather than a specific time
Info overload in appointments, device on you, set-up, and training. But it is all too much to take in	Understanding the data.	Data are initially taken in and monitored and viewed online by others
	Where does the data go?	Stressful for Noori to view it; so trying to set up so the data are seen by people who can be supportive and helpful
Desire to understand the exact process by which Andy came to be referred at the current time, with an SMI	Are the standard questions asked suitable for SMI to obtain a referral?	
For example, Annual diabetic review with practice, high HbA1c over 80; referred due to rise; and already on two or three types of medication. Then, answered a series of other questions?	What is the general framework and is SMI mentioned at all?	

Early days

**Table:** 

What happens during first two months?	Problems?	What helps solve the problem?
Missed appointments are more likely	Early wobble, has to go back for a second appointment to re-fit the device, and so not continuous data to start with	Drop-in clinic
Cann't cope with the data overload	CGM shows that his alcohol may need support with that. He need help dealing with the addiction issues	Call once every couple of weeks to check-in
Stressful to go to appointments by himself	Support with the ongoing monitoring aspects	Continuity is important with people with bipolar disorder, so it helps if the same people provide the care
Trust is established in the early days. Until that point, Andy attends appointments with another person		

Ongoing

**Table:** 

What happens during subsequent months and years?	Problems?	What helps solve the problem?
When feeling upset needs someone to contact		Good relationship with nurse specialist
Needs more checkups than average		Has a call when needed
Information may sometimes sit with Noor or need to be in a report		Contact eases once got set-up

After the group work phase, we facilitated a whole-group discussion to gain insights into commonalities and differences among the small groups and to begin identifying potential priority candidate components.

#### Step 6: synthesis, prototyping, and preparation for the joint showcase

After the co-design session, the project team synthesised and integrated the outputs into a structured pathway, ready to be presented as a prototype candidate intervention at the final session.

We maintained the three-stage structure, adapting the terminology to better capture the language used by participants in the three phases of the prototype intervention: (1) set-up, (2) early days, and (3) living with CGM. We used the CGM journey notes to draw out candidate intervention components for each phase of the prototype intervention. We referred to the persona descriptions to integrate context and determine priority areas for candidate components to address. The prototype intervention was summarised in a slide deck that showed each candidate component. We developed a new persona to “test out” the new intervention and illustrate how it might work together to address identified barriers and provide facilitators. We prepared session materials to facilitate a prioritisation exercise to identify the most important intervention components.

As before, we used high-level qualitative analysis to organise our workshop notes into descriptive themes, which are reported as follows.

#### Step 7: joint showcase and celebration

In the final session of this co-design study, the project team once again worked in partnership with service users, carers, and HCPs to review the prototype intervention and expand on the content of the candidate intervention components. Like session 3, this was a hybrid session, organised and managed in the same way to ensure a good experience for participants.

We used the novel persona to illustrate the prototype intervention by providing an exemplar case study of a person with SMI and type 2 diabetes travelling through the newly designed intervention. At this stage, the candidate components were presented as optional offers of support during the set-up, the early days, and the living with CGM phases. Following this initial overview, participants were asked to work in small groups to “walk” the personas through the prototype pathway. They were also invited to indicate their one or two top priority candidate components for each phase (set-up, early days, living with CGM). Components were developed in more detail with an emphasis on understanding participants' reasons for prioritising some components but not others. Supplementary Appendix 5 summarises the outcome of the prioritisation exercise undertaken in this session.

#### Step 8: final synthesis and development of the logic model

After completing the four workshop sessions, the project team iteratively synthesised and refined all outputs. We made changes to the prototype intervention where indicated based on the conversations in session 4; for example, the discussion around reasonable adjustments was moved before the “yes/no” decision point, rather than after. We summarised and synthesised the details provided for each candidate intervention component. All components were considered, regardless of their prioritisation score, and we captured uncertainty or disagreement between participants in the detail for individual components, for example, preference for in-person or telephone appointments, to showcase the need for individually tailored approaches.

To produce the logic model, we synthesised all workshop outputs to create a list of essential *Inputs/resources* and then categorised workshop outputs into *activities* (plain language descriptions of the active ingredients of the intervention), *BCTs* (translating activity descriptions into theory informed language), and *MoAs* [using the Theory and Techniques Tool (47) to systematically describe the means by which the new intervention is expected to affect change] within the three stages of the intervention (set-up, early days, living with CGM). We populated columns for initial *outputs*, *short-term outcomes*, *long-term outcomes*, and *impact* to capture the hypothesised effect of the new intervention, along with measurable milestones, over time (48). This was undertaken by JB, discussed with the other co-authors, and visually presented by JH.

To ensure that the logic model and programme theory were contextually relevant to the barriers and facilitators identified and discussed in the workshops, we undertook a high-level qualitative analysis of our workshop notes to draw out narrative themes across the workshops and how they correspond to the individual intervention components. This qualitatively captures the essence of the new intervention in ways that the structured logic model and programme theory cannot. All qualitative analyses were performed by JB. PC and IK sense checked and revised the identified themes. We produced a final version of all project outputs in an accessible language and shared this with participants.

### Decision making priorities and guiding principles

Throughout the project, we aimed to prioritise service user and carer perspectives. Where there were differences of opinion or preference between participants, we captured these in the development of the candidate components with the potential for refinement in future evaluations. During the co-design sessions, we made a conscious effort to enable different perspectives to be shared and discussed, with the aim of understanding the underlying reasons or circumstances for any disagreements.

### Responsible research and innovation

Supplementary Appendix 6 summarises our consideration of equity, diversity, and inclusion in this project, as well as reflections on participant burden and our approach to participant recognition.

#### Study management

Information about study management, governance, and data protection, as well as participant and researcher safety and wellbeing are provided in Supplementary Appendix 6.

## Results

This study aimed to co-design a logic model and programme theory for a new structured CGM intervention for people with SMI and type 2 diabetes. To do this, we conducted a four-step co-design study to identify barriers and facilitators and to develop the structure and content of the intervention.

### Participants

Ten participants took part in the study:

- Four HCPs
  ○ One GP
  ○ One primary care practice nurse
  ○ Two mental health practitioners
- Two informal carers
- Four service users

Six participants identified as male, and 9/ of the 10 were White. Age was not recorded.

Two of the HCPs and two of the service users were involved in the DIAMONDS feasibility study, while one service user is a current user of the FreeStyle Libre CGM system. Both carers and three of the service users were actively involved in different research projects relating to physical and mental illness. Participants were able to create rich histories for the personas, drawing on their own professional and/or personal experiences of the challenges associated with diabetes management in the context of co-existing SMI.

### Session attendance

Table 2 summarises the attendance at each session.

**Table 2:** Session attendance.

Session	Number of HCPs attending	Number of service users attending	Number of carers attending	Total attendees	Team members
1: HCP discovery session	4	N/A	N/A	4	JB, IK, JH, OM
2: Service user and carer discovery session	N/A	2	1	3	JB, IK, JH, OM
3: Joint co-design session	3	3	1	7	JB, IK, JH, OM
4: Final showcase and celebration	2	4	2	8	JB, IK, JH

### Qualitative workshop findings

After each round of workshops, we undertook a high-level qualitative analysis to summarise workshop outputs into descriptive themes. This provided important context for the development of the intervention and its subsequent logic model and offered a way to capture participant priorities.

#### Discovery sessions

Broadly, we identified four themes from our workshop notes: (1) the emotional impact of diabetes management, (2) the role of CGM, (3) family and social dynamics, and (4) intersectionality with broader life factors, including overall health and wellbeing, diet, and physical activity. The sense from the notes was that managing diabetes takes a significant emotional toll, with poor management worsening anxiety and depression, creating a cycle that hinders self-care. This strain extends to carers, who often take on extra responsibilities, leading to stress and family tension. While CGM sensors provide valuable real-time data, they can also cause stress. In addition, diabetes management is often complicated by pre-existing mental health issues.

#### Joint co-design event

Across the workshop, from the notes taken on the day, there were five common themes: (1) barriers to CGM access and systemic inefficiencies, (2) the need for personalised and integrated care, (3) support with technology and data, (4) ongoing education and encouragement, and (5) the role of relationships and equity.

These themes collectively emphasise the multifaceted nature of CGM adoption and management. Barriers like administrative delays and rigid systems hinder access, especially for vulnerable groups. Personalised care approaches are vital, integrating diabetes and mental health support to ensure holistic management. While powerful, technology and data require accessible solutions to reduce user stress and improve engagement. Sustainable behaviour change can be supported by ongoing education, practical advice, and positive reinforcement. Finally, strong relationships with caregivers and professionals, along with equitable access to resources, are essential to ensuring comprehensive and inclusive healthcare.

#### Joint showcase and final reflections

Six themes were identified from the discussions: (1) trust and communication, (2) personalisation and equity, (3) comfort and control, (4) support structures, (5) practicality in training and follow-ups, and (6) ongoing and integrated care. Table 3 shows how each of the candidate components reflects the identified themes, highlighting the coherence of the structured CGM intervention that was co-designed with participants in this study.

**Table 3:** Candidate components mapped to workshop themes.

Set-up		
Component number	Candidate component	Workshop theme(s)
1	Initial conversation	Trust and communication
		Personalisation and equity
2	Find out more	Personalisation and equity
		Comfort and control
		Support structures
4	Reasonable adjustments	Personalisation and equity
		Comfort and control
		Support structures
3	Yes/no	Trust and communication
		Comfort and control
5	1st set-up Appointment	Personalisation and equity
		Support structures
		Practicality in training and follow-ups

Early days		
Component number	Candidate component	Workshop theme(s)
6	Set-up follow up 1 week	Support structures
		Practicality in training
		Ongoing and integrated care
7	Set-up follow up 2 weeks	Support structures
		Practicality in training
		Ongoing and integrated care
8	Phone calls	Trust and communication
		Personalisation and equity
		Ongoing and integrated care
9	Additional appointments (including tech lesson)	Support structures
		Practicality in training
		Ongoing and integrated care
10	Sign-off appointment	Trust and communication
		Personalisation and equity
		Support structures

Ongoing support		
Component number	Candidate component	Workshop theme(s)
11	Support Group	Personalisation and equity
		Support structures
12	Regular calls/communication	Trust and communication
		Ongoing and integrated care
13	Relapse	Trust and communication
		Comfort and control
		Personalisation and equity
		Support structures
14	Additional appointments	Trust and communication
		Personalisation and equity
		Ongoing and integrated care
15	Annual review	Personalisation and equity
		Ongoing and integrated care

The six themes highlight the importance of a patient-centred approach in addressing the complex challenges faced by individuals managing both diabetes and SMI. They underline the necessity for diabetes care that is not only responsive to individual needs but also proactive in fostering trust, accessibility, and long-term engagement, for example, through continuity of care (i.e., seeing the same HCP each time) and through flexible, personalised support, including accommodation of a range of contact and appointment preferences (letters, phone calls, text messaging/email, in-person or remote consultations, care in the community, home visits, etc.). Support groups were a divisive subject, as was the reliance on remote consultations (by phone or video call), with some participants in favour and others against. Importantly, participants highlighted that they are likely to have different requirements and preferences for support depending on their overall physical and mental health and on wider circumstances in their lives. It was clear in the workshops that participants' confidence with technology varied widely and there was an expectation that the new CGM care pathway should be sufficiently flexible to meet these different needs, offering flexible and tailored support. The need for the HCPs involved in delivering this care pathway to have an understanding and appreciation of both mental and physical healthcare was highlighted and stressed repeatedly by all participants in the workshops. Addressing diabetes and possible lifestyle changes in isolation from the co-existing SMI is described as disempowering and frustrating by service users and carers and leads to an erosion of trust in the system. HCPs recognise gaps in their own and colleagues' training around the complexities of co-existing physical long-term conditions and SMI, and the impact this has on the delivery of care to patients.

The refined intervention was visualised and summarised in a logic model (see below). The visualisation, along with a summary report, was shared with participants within a month of the final session; this is included in Supplementary Appendix 7.

### Logic model and programme theory

**Figure 4:**
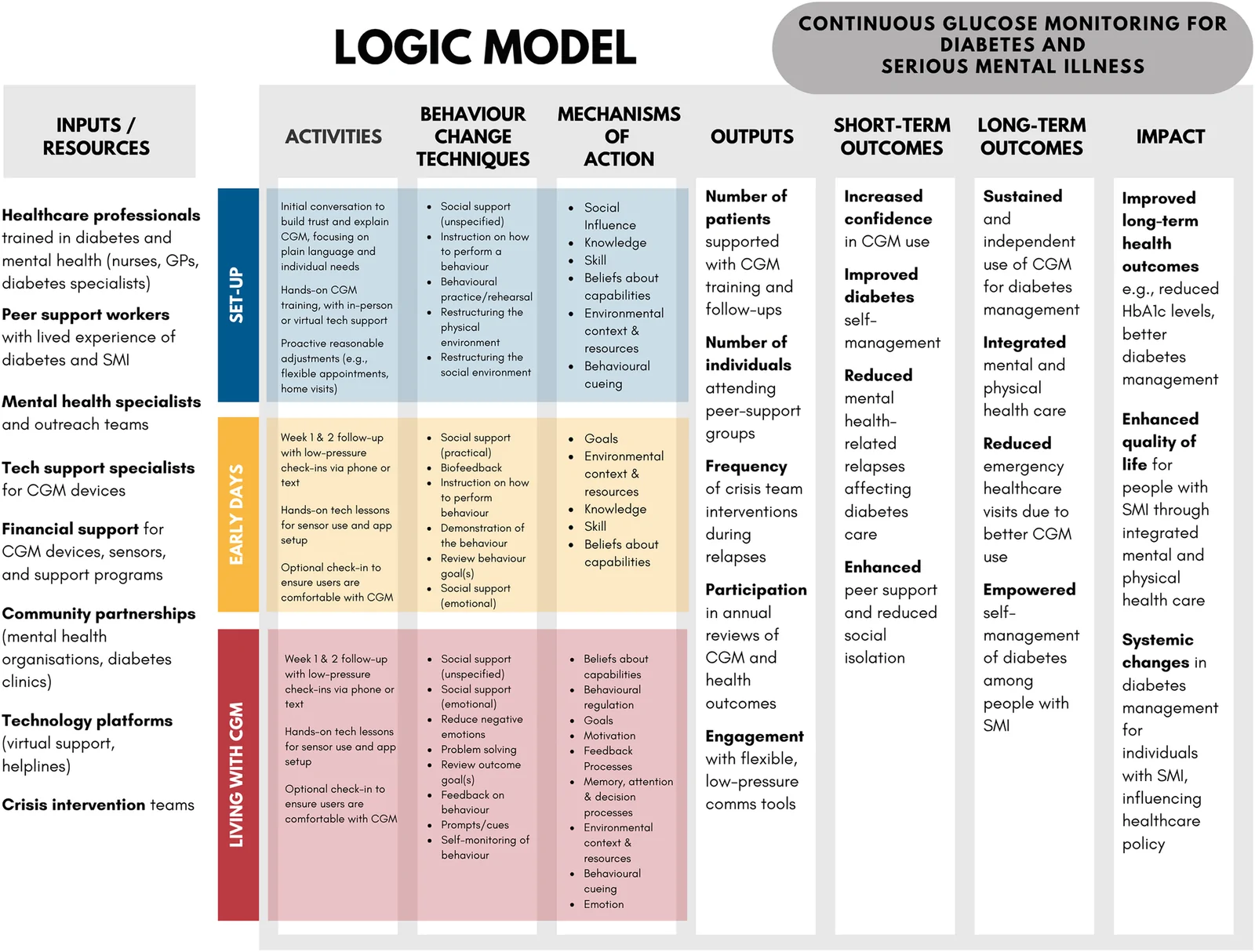
Logic model.

Figure 4 shows the final logic model summarising the intervention content that was developed throughout this co-design study.

#### Programme theory for CGM in diabetes and SMI

Programme theory, i.e., a description of how an intervention is expected or hypothesised to work, is a crucial part of intervention development (49). Based on the logic model presented in Figure 4, we developed the following programme theory to explain the interactions between the candidate components and the overall working structure of the intervention, which is summarised in the following.

#### Programme theory statement

By providing tailored technical, social, and emotional support for CGM, this intervention empowers individuals with diabetes and SMI to achieve improved diabetes self-management and overall health. With training, trust-building, and peer support, participants are more likely to maintain consistent CGM use, resulting in improved glucose control, reduced reliance on crisis interventions, and enhanced quality of life. In the long run, the programme serves as a model for integrated healthcare practices, with the potential to drive systemic change in diabetes management policies for people with complex health needs.

Supplementary Appendix 8 includes a completed TIDieR checklist (Template for Intervention Description and Replication) (50) based on the logic model and programme theory.

### Likely adaptations and remaining uncertainty

#### Certainty

Our study found that people with SMI and diabetes, their carers, and HCPs see a need for a structured CGM intervention tailored to this group. Co-design was vital to capture key perspectives. Service users emphasised the importance of integrating physical and mental healthcare, trusting HCP relationships, and flexible delivery to meet changing mental health needs. Practical support, such as recipes, shopping advice, prompts, encouragement, and accessible methods, was seen as beneficial for behaviour change.

#### Uncertainty

The intervention design is incomplete, with uncertainty about material design and implementation details such as location, personnel, and duration. Participants suggested printed materials and a familiar HCP contact, but further research must address costs and the best component combination.

#### Unknowns

The reception and effectiveness of this intervention in clinical practice are uncertain. Cultural adaptations will likely be needed to address health inequalities, but the specific cultures/languages affected, and the extent of the required changes remains unknown.

## Discussion

We worked with service users, carers, and HCPs; identified intervention components and their likely application to support the use of CGM; and then mapped these elements to a logic model to explain how it might work. This new structured intervention integrates mental and physical healthcare, provides ongoing support and education, and includes reasonable adjustments on a continuous opt-out basis.

Our findings demonstrate that a comprehensive logic model and programme theory can be developed rapidly, even with a population that is underrepresented in research and that may be considered “hard to reach.” The success of this rapid development was attributable to the structured, iterative nature of the accelerated co-design process and the use of digital tools that facilitated quick feedback cycles. In addition, we drew on our experience working with people with SMI and approached our work in ways that were respectful of their needs, such as offering support with travel booking, using plain language throughout, and sending session reminders. Online and hybrid meetings were effective, with dedicated facilitators ensuring inclusion.

A key strength of our approach is the explicit integration of behaviour change theory. The components prioritised by participants, such as the need for an “Initial Conversation” and ongoing support, map directly onto established MoAs and BCTs are detailed in our logic model. For instance, the high priority given to building a trusted relationship with an HCP reflects the BCT of “Social support (emotional)” and fosters the MoA of “Beliefs about capabilities” (also known as self-efficacy) by building confidence. Similarly, the desire for hands-on tech lessons and follow-up directly corresponds to BCTs such as “Instruction on how to perform a behaviour,” enhancing participants' skills and self-efficacy. This demonstrates that the needs identified through co-design align with evidence-based principles of behaviour change.

The perceived importance of these key elements, shared by participating service users, carers, and HCPs, clearly highlights where mainstream diabetes education programmes, like DESMOND, do not meet the needs of the population with co-existing SMI and type 2 diabetes. For example, the need for tailored support over time, the specific focus on mental health, and the importance of reasonable adjustments to increase accessibility are not covered by existing programmes, including freely available CGM support online, such as materials provided by device manufacturers.

SMI-specific research on CGM use remains limited. A study of 279 adults with type 2 diabetes found a 1.4% prevalence of schizophrenia or bipolar disorder, with no reported association between SMI and glycaemic metrics (51). A recent mixed-methods study conducted in Denmark on people with SMI and type 2 diabetes showed high acceptability of CGM among users and clinicians, as well as good integration into care pathways at a specialist diabetes clinic (52). Some of the challenges identified in the qualitative work mirror our findings reported here, suggesting some transferability across settings. Current CGM research rarely accounts for SMI, despite growing recognition that behavioural and contextual factors are critical in diabetes care. Some researchers suggest integrating behavioural, psychological, and glycaemic data to enhance precision medicine (53), though feasibility remains uncertain due to sedentary lifestyles (54) and potential concerns over continuous monitoring (55).

Recent trials exploring integrated digital healthcare platforms indicate potential benefits for glycaemic control and weight loss (56). However, the complexity of the intervention makes it difficult to isolate effective components. Future research should prioritise equity, personalisation, health service integration, and rigorous evaluation (57), aligning with our study's objectives.

Our findings highlight the urgent need for targeted research in collaboration with individuals with SMI. With further refinement, this intervention could address health inequalities by improving diabetes care. Expanding CGM access could mitigate socioeconomic disparities, as evidenced by research showing its impact on glycaemic control in children from deprived areas (54). Investigating similar effects in adults with SMI is crucial for reducing mortality gaps (55, 56).

### Limitations and generalisability

Our sample size was small (*N* = 10), though in line with comparable studies. It is important to acknowledge that we could not capture a wide variety of views and perspectives in this study, which may limit the generalisability of our findings. Most service user participants were White. While this approximates the demographic make-up of the people with diagnosed SMI in the United Kingdom (due to lower rates of diagnosis in other Ethnic groups), it means that we could not consider different cultural needs. This should be a priority area for future studies of this kind. People who face the intersectionality of living with SMI and being part of a minoritised Ethnic group are likely to have considerably worse outcomes, making further research in this area crucially important to address persistent health inequalities. While people with SMI are in and of themselves a persistently underserved group, regardless of Ethnicity, it is important not to lose sight of further layers of inequality within this group.

We also worked with only a small number of HCPs. We aimed to include a range of job roles and were able to capture some key ones but wider engagement from across the healthcare system would strengthen their perspective. This should be a key consideration for future work, especially for those with a focus on implementation.

Furthermore, it is important to note that all participants had some previous exposure to research and were known to the study team from previous work. This may mean that they are not representative of the wider population with SMI who tend to find it difficult to engage with services and are often distrustful of research and researchers. This needs to be kept in mind in future research to ensure interventions are accessible to all people with SMI, not just those who find it easy to engage.

The logic model and programme theory are largely specific to the context of our study—i.e., the UK healthcare system and the particular intersection of SMI and type 2 diabetes—but the qualitative findings highlighting service user and carer priorities are generalisable to other settings. Our approach also highlights that rapid co-design is possible with a population often considered “hard to reach,” provided the right support is offered. This is true universally and across contexts.

## Conclusions

We have developed a logic model and programme theory for a structured CGM intervention for people with SMI and a type 2 diabetes diagnosis, highlighting the importance of joined-up mental and physical healthcare, flexible support, and reasonable adjustments offered as standard. Safety netting during a mental health exacerbation was highlighted as particularly important.

The logic model and programme theory provide a theory- and evidence-based foundation for the further development of the intervention components, ready for evaluation in future research.

## Data Availability

The raw data supporting the conclusions of this article will be made available by the authors, without undue reservation.

## References

[1] De Hert M Dekker JM Wood D Kahl KG Holt RIG Möller HJ. Cardiovascular disease and diabetes in people with severe mental illness. Position statement from the European Psychiatric Association (EPA), supported by the European Association for the Study of Diabetes (EASD) and the European Society of Cardiology (ESC). Eur Psychiatry. (2009) 24:412–24. 10.1016/j.eurpsy.2009.01.005 19682863

[2] Reilly S Olier I Planner C Doran T Reeves D Ashcroft DM. Inequalities in physical comorbidity: a longitudinal comparative cohort study of people with severe mental illness in the UK. BMJ Open. (2015) 5(12):e009010. 10.1136/bmjopen-2015-009010 26671955 PMC4679912

[3] Stubbs B Vancampfort D De Hert M Mitchell AJ. The prevalence and predictors of type two diabetes mellitus in people with schizophrenia: a systematic review and comparative meta-analysis. Acta Psychiatr Scand. (2015) 132(2):144–57. 10.1111/acps.12439 25943829

[4] Scheuer SH Kosjerina V Lindekilde N Pouwer F Carstensen B Jørgensen ME. Severe mental illness and the risk of diabetes complications: a nationwide, register-based cohort study. J Clin Endocrinol Metab. (2022) 107(8):e3504–14. 10.1210/clinem/dgac204 35359003

[5] National Mental Health Intelligence Network. Premature mortality in adults with severe mental illness (SMI) (2023). https://www.gov.uk/government/publications/premature-mortality-in-adults-with-severe-mental-illness/premature-mortality-in-adults-with-severe-mental-illness-smi

[6] Siddiqi N Doran T Prady SL Taylor J. Closing the mortality gap for severe mental illness: are we going in the right direction? Br J Psychiatry. (2017) 211(3):130–1. 10.1192/bjp.bp.117.203026 28864752

[7] Harris JI Hanson D Leskela J Billig J Padilla-Martinez V Boyd J. Reconsidering research exclusion for serious mental illness: ethical principles, current status, and recommendations. J Psychiatr Res. (2021) 143:138–43. 34487990 10.1016/j.jpsychires.2021.09.016

[8] Coxon A McBain H Pavlova N Rowlands H Mulligan K. Are diabetes self-management programmes for the general diabetes population effective for people with severe mental illness?: a systematic review. BMC Psychiatry. (2020) 20(1):386. 10.1186/s12888-020-02779-7 32711492 PMC7382073

[9] Tranberg K Due TD Rozing M Jønsson ABR Kousgaard MB Møller A. Challenges in reaching patients with severe mental illness for trials in general practice—a convergent mixed methods study based on the SOFIA pilot trial. Pilot Feasibility Stud. (2023) 9(1):182. 10.1186/s12888-020-02779-7 doi: 10.1186/s40814-023-01395-y 37908003 PMC10617218

[10] Kanuch SW Cassidy KA Dawson NV Athey M Fuentes-Casiano E Sajatovic M. Recruiting and retaining individuals with serious mental illness and diabetes in clinical research: lessons learned from a randomized, controlled trial. J Health Dispar Res Pract. (2016) 9(3):115–26. 28533944 PMC5438211

[11] Mercer SW Gunn J Bower P Wyke S Guthrie B. Managing patients with mental and physical multimorbidity. BMJ Br Med J. (2012) 345:e5559. 10.1136/bmj.e5559 22945951

[12] Whitehorne-Smith P Lalwani K Martin R Mitchell G Milbourn B Abel W. A grounded theory exploration of the enablers and barriers of public healthcare access for people with comorbid serious mental and chronic physical illnesses in Jamaica. PLoS One. (2024) 19(8):e0309678. 10.1371/journal.pone.0309678 39213323 PMC11364246

[13] Zabell V Arnfred SM Rønne ST Berring LL Lerbæk B Jørgensen R. Combining diabetes and mental health care: an ethnographic exploration of user involvement in combined care. J Clin Nurs. (2023) 32(17–18):6622–33. 10.1111/jocn.16703 37166281

[14] Bochicchio L Stefancic A McTavish C Tuda D Cabassa LJ. Being there” vs “being direct”: perspectives of persons with serious mental illness on receiving support with physical health from peer and non-peer providers. Adm Policy Ment Health. (2021) 48:539–50. 10.1007/s10488-020-01098-z 33479782 PMC8043989

[15] Lean M Fornells-Ambrojo M Milton A Lloyd-Evans B Harrison-Stewart B Yesufu-Udechuku A. Self-management interventions for people with severe mental illness: systematic review and meta-analysis. Br J Psychiatry. (2019) 214(5):260–8. 10.1007/s10488-020-01098-z doi: 10.1192/bjp.2019.54 30898177 PMC6499726

[16] Smith R Han L Ali S Prady SL Taylor J Hughes T. Glucose, cholesterol and blood pressure in type II diabetes: a longitudinal observational study comparing patients with and without severe mental illness. J Psychiatr Ment Health Nurs. (2019) 26(9–10):347–57. 31287193 10.1111/jpm.12546

[17] Wu L-C Lai CY Huang C-J Chou FH-C Yu ET Yu C-Y. Psychological distress and diabetes self-management in patients with type 2 diabetes and comorbid serious mental illness. Arch Psychiatr Nurs. (2020) 34(4):218–23. 10.1016/j.apnu.2020.04.013 32828352

[18] Taheri Z Tanha Z Amraee K Hassanvandi S. Determining the causal relationships between the brain-behavioral system and psychological vulnerability in female patients with diabetes based on the mediator role of anxiety sensitivity. J Nurs Educ. (2023) 12(4):44–55. 10.22034/JNE.12.4.44

[19] Leicester Diabetes Centre at University Hospitals of Leicester NHS Trust. DESMOND (2020). Available online at: https://www.desmond.nhs.uk/ (accessed November 27, 2023).

[20] Roberts SH Bailey JE. An ethnographic study of the incentives and barriers to lifestyle interventions for people with severe mental illness. J Adv Nurs. (2013) 69(11):2514–24. 10.1111/jan.12136 23621276

[21] Aschbrenner K Carpenter-Song E Mueser K Kinney A Pratt S Bartels S. A qualitative study of social facilitators and barriers to health behavior change among persons with serious mental illness. Community Ment Health J. (2013) 49(2):207–12. 10.1111/jan.12136 doi: 10.1007/s10597-012-9552-8 23054155

[22] Polonsky WH Fisher L. When does personalized feedback make a difference? A narrative review of recent findings and their implications for promoting better diabetes self-care. Curr Diab Rep. (2015) 15(8):50. 10.1007/s11892-015-0620-7 26077015

[23] Murphy JF Amin LB Celikkaleli ST Brown HE Tapan U. Disparities in cancer care in individuals with severe mental illness: a narrative review. Cancer Epidemiol. (2024) 93:102663. 10.1016/j.canep.2024.102663 39255550

[24] Mulligan K McBain H Lamontagne-Godwin F Chapman J Haddad M Jones J. Barriers and enablers of type 2 diabetes self-management in people with severe mental illness. Health Expect. (2017) 20(5):1020–30. 10.1111/hex.12543 doi: 10.1016/j.canep.2024.102663 28306182 PMC5600230

[25] Richardson KM Jospe MR Bohlen LC Crawshaw J Saleh AA Schembre SM. The efficacy of using continuous glucose monitoring as a behaviour change tool in populations with and without diabetes: a systematic review and meta-analysis of randomised controlled trials. Int J Behav Nutr Phys Act. (2024) 21(1):145. 10.1186/s12966-024-01692-6 39716288 PMC11668089

[26] Polonsky WH Fortmann AL Soriano EC Guzman SJ Funnell MM. The AH-HA! project: transforming group diabetes self-management education through the addition of flash glucose monitoring. Diabetes Technol Ther. (2023) 25(3):194–200. 36409486 10.1089/dia.2022.0419

[27] National Institute for Health and Clinical Excellence (NICE). Guideline: type 1 diabetes in adults: diagnosis and management. Draft for consultation 2021. Available online at: https://www.nice.org.uk/guidance/GID-NG10265/documents/draft-guideline (accessed December 5, 2021).

[28] National Institute for Health and Clinical Excellence (NICE). Guideline: type 2 diabetes in adults: management. Draft for consultation 2021. Available online at: https://www.nice.org.uk/guidance/GID-NG10264/documents/draft-guideline (accessed December 5, 2021).

[29] Zheng Y Campbell Rice B Melkus GD’E Sun M Zweig S Jia W. Dietary self-management using mobile health technology for adults with type 2 diabetes: a scoping review. J Diabetes Sci Technol. (2023) 17(5):1212–25. 10.1177/19322968231174038 37162011 PMC10563537

[30] Barnes TRE MacCabe JH Kane JM Delgado O Paton C. The physical health and side-effect monitoring of patients prescribed clozapine: data from a clinical audit conducted in UK mental health services. Ther Adv Psychopharmacol. (2020) 10:2045125320937908. 10.1177/2045125320937908 32821377 PMC7412922

[31] McBain H Mulligan K Haddad M Flood C Jones J Simpson A. Self management interventions for type 2 diabetes in adult people with severe mental illness. Cochrane Database Syst Rev. (2016) 2016(4):CD011361. 10.1002/14651858.CD011361.pub2 PMC10201333 27120555

[32] Brown JVE Coventry PA Siddiqi N Ajjan RA. 59th EASD annual meeting of the European Association for the Study of Diabetes. Diabetologia. (2023) 66(1):1–536. 10.1007/s00125-023-05969-6

[33] Michie S Van Stralen MM West R. The behaviour change wheel: a new method for characterising and designing behaviour change interventions. Implement Sci. (2011) 6(1):42. 10.1186/1748-5908-6-42 21513547 PMC3096582

[34] Cane J O’Connor D Michie S. Validation of the theoretical domains framework for use in behaviour change and implementation research. Implement Sci. (2012) 7:1–17. 10.1186/1748-5908-7-37 PMC3483008 22530986

[35] Michie S Richardson M Johnston M Abraham C Francis J Hardeman W. The behavior change technique taxonomy (v1) of 93 hierarchically clustered techniques: building an international consensus for the reporting of behavior change interventions. Ann Behav Med. (2013) 46(1):81–95. 10.1007/s12160-013-9486-6 23512568

[36] Richardson M Khouja CL Sutcliffe K Thomas J. Using the theoretical domains framework and the behavioural change wheel in an overarching synthesis of systematic reviews. BMJ Open. (2019) 9(6):e024950. 10.1136/bmjopen-2018-024950 31229999 PMC6596985

[37] Wight D Wimbush E Jepson R Doi L. Six steps in quality intervention development (6SQuID). J Epidemiol Community Health. (2016) 70(5):520–5. 10.1136/jech-2015-205952 26573236 PMC4853546

[38] O’Cathain A Croot L Duncan E Rousseau N Sworn K Turner KM. Guidance on how to develop complex interventions to improve health and healthcare. BMJ Open. (2019) 9(8):e029954. 10.1136/bmjopen-2019-029954 PMC6701588 31420394

[39] Moore G Campbell M Copeland L Craig P Movsisyan A Hoddinott P. Adapting interventions to new contexts—the ADAPT guidance. Br Med J. (2021) 374:n1679. 10.1136/bmj.n1679 34344699 PMC8329746

[40] Duncan E O'Cathain A Rousseau N Croot L Sworn K Turner KM. Guidance for reporting intervention development studies in health research (GUIDED): an evidence-based consensus study. BMJ Open. (2020) 10(4):e033516. 10.1136/bmjopen-2019-033516 32273313 PMC7245409

[41] Locock L Robert G Boaz A Vougioukalou S Shuldham C Fielden J. Health Services and Delivery Research. Testing Accelerated Experience-Based CC-Design: A Qualitative Study of Using a National Archive of Patient Experience Narrative Interviews to Promote Rapid Patient-Centred Service Improvement. Southampton, UK: NIHR Journals Library (2014). 25642558

[42] Grindell C Coates E Croot L O’Cathain A. The use of co-production, co-design and co-creation to mobilise knowledge in the management of health conditions: a systematic review. BMC Health Serv Res. (2022) 22(1):877. 10.1186/s12913-022-08079-y 35799251 PMC9264579

[43] Clarke D Gombert-Waldron K Honey S Cloud G Harris R Macdonald A. Co-designing organisational improvements and interventions to increase inpatient activity in four stroke units in England: a mixed-methods process evaluation using normalisation process theory. BMJ Open. (2021) 11(1):e042723. 10.1136/bmjopen-2020-042723 33500286 PMC7839845

[44] The King's Fund. Who are unpaid carers, who are they and how often do they provide care? (2024). https://www.kingsfund.org.uk/insight-and-analysis/data-and-charts/unpaid-carers-nutshell

[45] Dominello A Cheek C Clay-Williams R. The use of personas to involve consumers in healthcare co-design: a scoping review. BMC Health Serv Res. (2025) 25(1):649. 40329348 10.1186/s12913-025-12786-7 PMC12054091

[46] Davies EL Bulto LN Walsh A Pollock D Langton VM Laing RE. Reporting and conducting patient journey mapping research in healthcare: a scoping review. J Adv Nurs. (2023) 79(1):83–100. 10.1111/jan.15479 36330555 PMC10099758

[47] Johnston M Carey RN Connell Bohlen LE Johnston DW Rothman AJ De Bruin M. Development of an online tool for linking behavior change techniques and mechanisms of action based on triangulation of findings from literature synthesis and expert consensus. Transl Behav Med. (2021) 11(5):1049–65. 32749460 10.1093/tbm/ibaa050 PMC8158171

[48] Mills T Lawton R Sheard L. Advancing complexity science in healthcare research: the logic of logic models. BMC Med Res Methodol. (2019) 19(1):55. 10.1186/s12874-019-0701-4 30871474 PMC6419426

[49] Skivington K Matthews L Simpson SA Craig P Baird J Blazeby JM. A new framework for developing and evaluating complex interventions: update of medical research council guidance. Br Med J. (2021) 374:n2061. 10.1136/bmj.n2061 34593508 PMC8482308

[50] Hoffmann TC Glasziou PP Boutron I Milne R Perera R Moher D. Better reporting of interventions: template for intervention description and replication (TIDieR) checklist and guide. BMJ Br Med J. (2014) 348:g1687. 10.1136/bmj.g1687 24609605

[51] Chiang JI Manski-Nankervis J-A Thuraisingam S Jenkins A O'Neal D Mair FS. Multimorbidity, glycaemic variability and time in target range in people with type 2 diabetes: a baseline analysis of the GP-OSMOTIC trial. Diabetes Res Clin Pract. (2020) 169:108451. 10.1016/j.diabres.2020.108451 32949650

[52] Hansen AB Zabell V Juel A Berring LL Tarnow L Austin SF. Flash glucose monitoring and continuous glucose monitoring among patients with coexisting diabetes and severe mental illness—a convergent mixed-methods study. Nord J Psychiatry. (2025) 79(7):494–502. 10.1080/08039488.2025.2530629 40692370

[53] Hermanns N Ehrmann D Shapira A Kulzer B Schmitt A Laffel L. Coordination of glucose monitoring, self-care behaviour and mental health: achieving precision monitoring in diabetes. Diabetologia. (2022) 65(11):1883–94. 10.1007/s00125-022-05685-7 35380233 PMC9522821

[54] Bort-Roig J Briones-Buixassa L Felez-Nobrega M Guàrdia-Sancho A Sitjà-Rabert M Puig-Ribera A. Sedentary behaviour associations with health outcomes in people with severe mental illness: a systematic review. Eur J Public Health. (2020) 30(1):150–7. 10.1093/eurpub/ckz016 30793737

[55] Brunette MF Achtyes E Pratt S Stilwell K Opperman M Guarino S. Use of smartphones, computers and social media among people with SMI: opportunity for intervention. Community Ment Health J. (2019) 55(6):973–8. 10.1007/s10597-019-00431-7 31175518 PMC7534532

[56] Lee Y-B Kim G Jun JE Park H Lee WJ Hwang Y-C. An integrated digital health care platform for diabetes management with AI-based dietary management: 48-week results from a randomized controlled trial. Diabetes Care. (2023) 46(5):959–66. 10.2337/dc22-1929 36821833

[57] Kahkoska AR Cristello Sarteau A Crowley MJ. Delivering on the promise of technology to augment behavioral interventions in type 2 diabetes. Diabetes Care. (2023) 46(5):918–20. 10.2337/dci23-0009 37185694

