# Supplementary material for: Supporting continuous glucose monitoring for people with serious mental illness and type 2 diabetes: a co-design study for a structured intervention
Source: Front Health Serv. 2026 Aug 20;6:1807689. doi: 10.3389/frhs.2026.1807689 (PMC13538416; doi:10.3389/frhs.2026.1807689)
Supplement: Supplementary file 1 [file Table1.docx]

# Appendix 1 – GUIDED Checklist

Checklist adapted from Duncan et al. (2020).

| **Item number** | **Item description** | **Location in manuscript where item is reported** |
| --- | --- | --- |
| 1 | Report the context for which the intervention will be developed. | Page 4/5 |
| 2 | Report the purpose of the intervention development process. | Page 6 |
| 3 | Report the target population for the intervention development process. | Page 6 |
| 4 | Report how any published intervention development approach will contribute to the development process. | Page 7/8 |
| 5 | Report how evidence from different sources will inform the intervention development process. | Page 10 |
| 6 | Report how/if published theory will inform the intervention development process. | Page 5/6 |
| 7 | Report any planned use of components from an existing intervention in the current intervention development process. | Page 5 |
| 8 | Report any guiding principles, people, or factors that will be prioritised when making decisions during the intervention development process. | Page 17 |
| 9 | Report how stakeholders will contribute to the intervention development process. | Page 7 |
| 10 | Report how the intervention changed in content and format from the start of the intervention development process. | N/A |
| 11 | Report any changes to intervention required or likely to be required for subgroups. | N/A |
| 12 | Report important uncertainties at the end of the intervention development process. | N/A |
| 13 | Follow TIDieR guidance when describing the developed intervention. | N/A |
| 14 | Report the intervention development process in an open access format. | N/A |

# Appendix 2 – Recruitment and consent procedures, including participant-facing documents

## Service-user recruitment

We recruited service-users from existing cohorts of previous participants who have given consent to be recontacted about future research opportunities. All potential participants received an information pack about the study, including an invitation letter, participant information sheet, and consent form. We then contacted potential participants via telephone or email to discuss the study and to establish if they would be interested in taking part. During the phone call/in the email, it was emphasised that participation would involve attendance of three workshops over several weeks.

Those who confirmed their interest in taking part received an invitation to the first session by email as well as reminders via telephone call, text message, and/or email, as per their preference.

## Carer recruitment

We used the King’s Fund definition(49) of informal carers which has been adopted by the NHS and Department of Health and Social Care and includes anyone looking after “*a relative or friend who needs support because of … illness, including mental illness*”, offering “*active support, supervision, or social interaction*”. Carer participants could be part of a participating carer-and-service-user dyad or participate in the study on their own.

We asked service-user participants to identify informal carers, however, this did not yield any success. Therefore, carers were recruited using existing cohorts of carer-participants who have previously taken part in DIAMONDS and/or related studies and who have given consent to be contacted again about opportunities to take part in research. The process was identical to the one described above for service-users.

## Healthcare professional (HCP) recruitment

HCPs were recruited from existing networks and contacts, using a snowballing. We did not restrict participation by role, job title, or seniority.

HCPs were initially approached informally by email. If they expressed interest in the study, they received an invitation letter, information sheet, and consent form via email and were be asked to return the completed and signed consent form.

Once HCPs had confirmed their participation in the study, they received a written invitation to the first session via email as well as an email reminder nearer the time.

## Participant-facing documents

The following pages include the invitation letter, participant information sheet, and consent form used in this study. The version shown here was used for service users, adapted versions of the same documents were used for carers and HCPs.

Jennifer Brown

Mental Health & Addiction Research Group

Department of Health Sciences

University of York

YO10 5DD

Tel: 01904 321662 or 07384 239004

Email: jennifer.brown@york.ac.uk

[Name and address of participant]

[Date]

Dear [first name],

**Invitation to take part in a research study**

I would like to invite you to take part in an exciting new study to work with other people like yourself and with carers, healthcare staff, and researchers. Together, I would like to develop a new support programme for people with serious mental illness to help them manage their type 2 diabetes using technology called continuous glucose monitoring.

I am looking for people like yourself to join the study and share their experiences and ideas.

I have enclosed a Participant Information Sheet which gives more information about what is involved. A Consent Form is also included for your information.

I will call you in the next couple of days to see if you would like to take part. I will be happy to answer any questions you may have at that point.

I look forward to speaking to you soon, but if you have any questions in the meantime, please contact me using the details at the top of the page.

Thank you!

Yours sincerely,

Jennifer Brown

University of York

**Participant Information Sheet**

Designing a new support programme to help people with serious mental illness manage their type 2 diabetes

Thank you for reading this information sheet and for your interest in my project.

You are invited to take part in an exciting new study to work with other people like yourself and with carers, healthcare staff, and researchers. I want to design a new support programme for people with serious mental illness to help them manage their type 2 diabetes using technology called continuous glucose monitoring.

Before you decide if you want to take part, please take time to read the following information carefully.

- You are being asked to share your ideas and experience in groups with other people.
- This will involve three in-person meetings in York (or another city in the local area), each lasting about three hours. I will pay for your travel and provide refreshments.
- You will be offered a payment of £25 per hour for each meeting.
- Other people at the meetings will include doctors and nurses, researchers, carers, and others living with type 2 diabetes and serious mental illness.
- During the meetings, I will take photos and make notes. You do not have to be in any photos if you don’t want to. I will treat all the information you share with me in confidence.
- If you would like to take part, you will be asked to complete a consent form. A consent form is a written record that you’re happy to take part in the study. Please read the one that is included with this letter.
- You do not have to take part and you can stop taking part at any time without giving a reason. Your decision about whether or not to take part will not have an impact on the care you receive.
- **You do not need to do anything at this stage. I will contact you in the next few days to check if you would like to take part.**

The following pages explain the study in more detail and give you important information about taking part.

How to get in touch	Contents

If you have any questions about the study, you can contact me:

Jennifer Brown

Tel:	01904 321662

Mobile:	07384 239004

Email:	[jennifer.brown@york.ac.uk](mailto:jennifer.brown@york.ac.uk)

There is an answering machine available 24 hours a day, so please leave a message and I will return your call as soon as possible.

1 Why am I being asked to take part?
2 What will taking part involve?
3 Do I have to take part?
4 Possible benefits and disadvantages of taking part
5 Will the information I provide be confidential?
6 More information about taking part
7 What do I need to do now?
9 What do I need to do now?

Why am I being asked to take part?

1

I am looking to work with people like yourself who have serious mental illness and type 2 diabetes. I know that it can be difficult to stay well and live healthily. **Together, we can to develop a new way to manage type 2 diabetes that uses technology called continuous glucose monitoring.**

Continuous glucose monitoring is done by a small sensor that is attached to a person’s upper arm. It stays in place for two weeks. The sensor measures the person’s sugar levels all through the day and night. Understanding sugar levels is an important part of managing diabetes.

My previous work has shown that people with serious mental illness are happy to wear these sensors and get on well with them. **What I don’t yet know is how best to support individuals and their carers to get the most out of the new technology.**

You are being asked to take part in this study because you have previously taken part in research about serious mental illness and diabetes at the University of York. You gave permission to be contacted again about similar projects.

If you have a carer or someone who helps you with your day-to-day life, I may contact them separately to see if they would like to take part to share their experiences and views.

What will taking part involve?

2

**What happens next?**

**I will contact you by telephone call in the next couple of days. You will have a chance to ask any questions you have about taking part.**

I will then ask you if you would like to take part in the study.

If you do want to take part, I will let you know the date and time for the first meeting.

**If you do not want to take part, you will not have to do anything further.** I will not contact you again about this study.

**The meetings**

**I would like you to take part in three meetings. These will last for about three hours each.** The meetings will take place in an a safe and accessible venue in York (or a city nearby). In total, I am asking for about nine hours of your time for the meetings, plus any time it takes you to travel to the meetings.

I can pay for your travel costs to come to the meetings. If you need childcare or other support to take part, I can pay for that, as well. If you prefer, you are welcome to have a family member, friend, or carer with you during the meetings.

There will be one meeting every couple of weeks. I will let you know by letter and phone call when each meeting will take place. I will also send you reminders nearer the time.

For the study, it is important that there are enough people at each meeting. **I ask you to commit to coming to at least two out of the three meetings.**

However, if you change your mind at any point or you are no longer available, you can let me know. **You will not have to take part if you don’t want to and you don’t have to tell me why.**

At the meetings, I will be supported by a team of colleagues, including a facilitator who will lead the meetings. The meetings will be relaxed and informal. Everyone will be given a chance to speak. There will be no right or wrong answers. We will make sure that everyone feels safe to share their experiences and ideas. Everything that is said will be treated in confidence.

**The 1^st^ meeting – Discovery session**

Before the first meeting I will go through the consent form with you. **You will be asked to sign the form to confirm that you understand the study and that you are happy to take part.** This is called “giving consent”. If you change your mind at this point, you will not have to give consent. You will not have to take part if you don’t want to.

**The first meeting is called a “discovery session”.** This meeting will be attended by people with serious mental illness and type 2 diabetes, like yourself, and by informal carers. Informal carers are family members or friends who support another person with their daily lives without receiving payment. There will be a separate discovery session for healthcare professionals like doctors and nurses.

At the discovery session, we will share some information with you about what it can be like for people with serious mental illness to try and manage their diabetes. We will also show you how the continuous glucose monitors work and what kind of information they collect.

We will ask you and the other people at the meeting to share your ideas about why it can be difficult to manage diabetes and how we can use the sensors to make this better.

**The 2^nd^ meeting – Co-design workshop**

You will receive an invitation to the second meeting by letter and phone call. You can let me know if you are available to attend.

**The second meeting will be a “co-design workshop”.** Everyone from the first meeting will be invited back. We will also ask healthcare professionals, such as doctors and nurses, to join the co-design workshop. Some of the healthcare professionals may join the meeting via video call.

Together, we will work in small groups to discuss everyone’s ideas from the discovery sessions. We will start developing a new support programme that includes continuous glucose monitoring.

**The 3^rd^ meeting – Celebration event**

You will receive an invitation to the third meeting by letter and phone call. You can let me know if you are available to attend. The third meeting will be the final meeting of the study.

**The third meeting will be called a “celebration event”.** During this meeting, we will look back over the work of the study and celebrate what we have achieved together. We will also think about what will happen next, for example further research that will be needed.

**What will I be asked to do at the meetings?**

At the meetings, we will ask you to take part in group activities. **This will involve talking about your experience of receiving healthcare and about things that you do in your day to day life.** You will not need to share anything that you feel uncomfortable about.

We will also ask you to share ideas and make suggestions about how you think we can provide better diabetes support for people with serious mental illness.

**To make sure that everyone can contribute, we will also ask you to listen to other people’s stories and ideas and to take on board what they are suggesting.**

For some activities, we may ask you to write things down or to use other materials to help us with the work. Everything will be explained on the day and you can ask questions at any point.

**After the meetings**

**After each meeting, you will be offered payment for taking part. We will offer you £25 per hour, that is £75 per meeting. This payment will be in addition to any travel or other expenses.**

You do not have to accept payment. **Before accepting payment, please check if this could affect any benefits you are receiving.**

If you have any questions between the meetings, you can contact me using the details on page 3.

Do I have to take part?

3

**You do not have to take part. You can change your mind about taking part at any point.**

If you do take part, you are free to stop at any time without giving a reason. Your decision will not affect any care you receive.

If you decide to stop taking part during a meeting, you can leave the meeting at any time. I will not be able to remove any contributions you have made to the meeting up to that point. **Your responses will still be used in the study even if you decide to stop taking part.**

If you decide to leave partway during one meeting, you are still welcome to return to the study for the next meeting if you like.

Benefits and disadvantages of taking part

4

**What are the possible benefits of taking part?**

I cannot promise that taking part will benefit you. However**, you will make an important contribution to the study by sharing your experiences and ideas.**

You will help me develop a new support programme for people with serious mental illness and diabetes. In future studies I will be able to test if this works. If it does, it may become available to people across the UK.

**What are the possible disadvantages of taking part?**

**Taking part will take up several hours of your time over a couple of months**. I will reimburse you for any expenses and will offer you payment for your time.

Will the information I provide be confidential?

5

**Your involvement in this study will be confidential and only members of the research team will know whether or not you have agreed to take part.** We will not inform your GP or any other healthcare providers about whether or not you have taken part.

**During the meetings we will record the discussions in photographs and by taking notes.** We will keep these records confidential and they will not be shared outside of the research team. You do not need to be in any photographs if you don’t want to.

**We will store the records from the meetings securely for a period of ten years after the end of the study. After this, they will be destroyed.**

I hope to share findings of the study in scientific journals and/or at conferences, as well as on a website and social media. I will not include your name or any information that could identify you.

6

More information about taking part

**What will happen to information collected about me during the study?**

**I will treat any information you give me in confidence. I will store all your information safely and securely. I will not mention your name in any publications about the study and I will make sure that no individuals can be identified in the study results.**

Sometimes researchers are asked to share information we collect in studies with colleagues who are looking at similar ways to improve healthcare. With your agreement, I would like to be able to share the information I collect about you as part of this study. I will always remove all identifiable information before I share your information. This is controlled by strict University of York and NHS Trust procedures.

I will keep the information you give me as part of the current study safely for ten years after the study ends. It will then be destroyed securely. Paper records will be handled only by the study team and kept in locked cabinets. Electronic records will be held on a secure computer server approved by the University of York. Only authorised members of the study team will have access to your information.

I will be happy to answer any questions you may have about how I use the information I receive from you as part of the study. Please contact me using the details on page 3.

**What if there is a problem?**

**If you have concerns about the study or if there is anything you would like to ask about the study, please get in touch.** My contact details are on page 3. You can also contact my supervisors if you have any concerns:

Prof Peter Coventry, [peter.coventry@york.ac.uk](mailto:peter.coventry@york.ac.uk)

Prof Najma Siddiqi, [najma.siddiqi@york.ac.uk](mailto:najma.siddiqi@york.ac.uk)

Prof Ramzi Ajjan, [r.ajjan@leeds.ac.uk](mailto:r.ajjan@leeds.ac.uk)

I do not expect that any harm will to come to you from being in this study.

If you wish to raise a complaint on how I have handled your personal data, you can contact the University of York’s Data Protection Officer who will investigate the matter (dataprotection@york.ac.uk). If you are not satisfied with my response or believe I am processing your personal data in a way that is not lawful you can complain to the Information Commissioner’s Office (ICO) whose details can be found here: https://ico.org.uk/.

**What will happen to the results of the study?**

**When the study has finished and the results have been published, I will send you a summary of the study results if you have agreed for me to do so.** You can contact me directly if you decide not to take part in the study but would still like to receive a summary of the results. I will also keep the study website up to date with news of the study.

**Who is organising and funding the study?**

I am doing this research as part of my PhD with the support of my supervision team based in the Department of Health Sciences at the University of York. I am not receiving payment to carry out this study but I have access to departmental and university funds to cover the costs of the research. In addition, this study is supported by a grant from the Institute for Mental Health Research at York (IMRY).

The study has been reviewed by an independent group of people, called a Research Ethics Committee. This is to protect your safety, rights, wellbeing, and dignity. This study was looked at and approved by the *[REC Details]* (Reference: *[Insert REC reference]*).

What do I need to do now?

7

**Right now, you don’t need to do anything.** You may want to discuss the information in this letter with a family member or friend. If you want, you can have a read of the enclosed consent form.

**I will contact you in a couple of days. You will have the chance to ask any questions you have about taking part. If you’re happy to go ahead, I will let you know the date and time for the first meeting.**

Thank you for taking the time to consider taking part in this study.

Data Protection statement

The University of York is the sponsor for this study based in the United Kingdom. We will be using information from you in order to undertake this study and will act as the data controller for this study. This means that we are responsible for looking after your information and using it properly.

The University of York is a publicly funded organisation that conducts research to improve health, care, and services. Research following the UK Policy Framework for Health and Social Care Research is conducted to serve the interests of society as a whole. This means that the University of York is using the legal basis provided under the Data Protection Act 2018 and the General Data Protection Regulation of ‘for the purposes of research conducted in the public interest’ to use your personal data for this research.

Your rights to access, change or move your information are limited, as we need to manage your information in specific ways in order for the research to be reliable and accurate. If you withdraw from the study, we will keep the information about you that we have already obtained. To safeguard your rights, we will use the minimum personally-identifiable information possible.

You can find out more about how we use your information at:

<https://www.york.ac.uk/healthsciences/research/trials/trials-gdpr/>

The University of York will use your name and contact details to contact you about the research study and to oversee the quality of the study. The people who analyse the information will not be able to identify you and will not be able to find out your name or contact details. Individuals from University of York and regulatory organisations may look at your research records to check the accuracy of the research study.

The University of York will keep consent forms and other information about you from this study for 10 years after the study has finished. Contact details will be kept for three years unless you have given consent to be contacted about future research.

**Thank you for reading this information.**

# Appendix 3 – Changes from the study protocol

The study protocol was registered on the Open Science Framework (OSF) on 2^nd^ February 2024 and can be accessed using the following link <https://osf.io/4ae3g/>. Further detail can also be found in the preprint available on MedRXiv: <https://www.medrxiv.org/content/10.1101/2024.05.16.24307473v1.full-text>

The table below summarises all instances where the actual study methods did not follow the approach described in the protocol along with reasons for those changes.

| **Planned approach as per the study protocol** | **Actual approach taken in the study** | **Reason for change** |
| --- | --- | --- |
| Recruitment of 15 participants | 10 participants took part in the study | Due to capacity and resource constraints, we were unable to recruit to our original target of 15 participants. Some individuals who had initially expressed an interest in taking part were unable to do so. We did not have time to continue ongoing recruitment. HCPs were unable to commit to attending multiple workshops.  We are confident that the smaller sample size did not negatively affect the rigour of the study as we were able to obtain in-depth data from the participants. Members of the research team contributed personal experience as carers and HCPs. |
| Step 1: Findings from a recent mixed-methods systematic review of the acceptability and feasibility of CGM in people with diabetes that have been analysed within the Theoretical Framework of Acceptability (TFA) will be interrogated further using the Theory and Techniques Tool linking BCTs and associated MoAs (i.e., short-term outcomes). In particular, we will extract detail from included studies that describe a structured CGM intervention, i.e. the use of “wrap-around” education and support. | We summarised top-level findings from the systematic review without the in-depth interrogation of “wrap-around” materials using BCTs and MoAs. | Due to delays in the completion of the systematic review, we needed to proceed with the co-design study before full review findings were available. Therefore, an in-depth interrogation of the findings and mapping against BCTs/MoAs was not possible. We did, however, summarise key headline findings about acceptability of CGM.  Taking a more top-level rather than in-depth approach to summarising the review findings allowed us to avoid undue delays to the co-design study. It also meant that we were able to deliver summary information concisely at the start of the co-design study without over-burdening participants with excess information. |
| Use of an animated catalyst film in the discovery sessions. | We introduced personas in the discovery sessions, using animations in subsequent workshops. | We adapted our approach for the discovery sessions, focusing on introducing personas rather than using an animated catalyst film. Participants responded well to this approach. Once personas had developed into detailed fictional characters, we developed animations for some of them to “bring their stories to life” in later workshop sessions. |
| Journey mapping, pain point analysis and prototyping in discovery sessions | The discovery sessions focused on the development of the personas. Journey mapping, pain point analysis, and prototyping were used in subsequent workshops. | We adjusted the pace of the discovery sessions to make room for free-flowing guided conversations and idea generation, rather than rigidly following structured methods. This met the needs of participants, especially people with SMI who appreciated the opportunity to talk freely and share their stories through the medium of persona development. |
| Step 4: Mapping against TFA, BCTs/MoAs, and refinement of the logic model. | Working with the outputs produced in the discovery sessions, we took a less structured approach to synthesis, working iteratively with the data and information available. | After successfully taking a less structured approach in the discovery sessions, we continued to work iteratively during the synthesis sessions that followed. This was in line with the outputs produced and paved the ground for further iterative and free-flowing development work in the following workshops. |
| Step 5: Focus on prioritising BCTs in joint co-design event. | Prioritisation was pushed back until the final workshop. | Due to the complexity inherent in the co-design of this structured intervention, driven largely by the complexities arising from co-existing SMI and physical health problems, we did not want to rush prioritisation of BCTs. Instead, we allowed more time for the generation and development of detailed intervention content to ensure we would gain a clear understanding of participants’ needs and preferences.  Formal prioritisation was completed as part of the final workshop. |

# Appendix 4 – Session materials

For convenience, session materials have been collated on the Open Science Framework (OSF), and can be accessed using the following link: [https://osf.io/4ae3g/files/osfstorage#](https://osf.io/4ae3g/files/osfstorage)

# Appendix 5 – Outcomes from prioritisation exercise

Table 1: Prioritisation ratings for each of the proposed intervention components during set up (blue), early days (yellow), and ongoing support (red).

| **Set up** | | |
| --- | --- | --- |
| 1 | Initial Conversation | 6 |
| 2 | Find out more | 3 |
| 4 | Reasonable Adjustments | 5 |
| 3 | Yes / No | 1 |
| 5 | 1st set-up Appointment | 6 |
| **Early days** | | |
| 6 | Set-up follow up 1 Week | 3 |
| 7 | Set-up follow up 2 Week | 4 |
| 8 | Phone calls | 2 |
| 9 | Additional appointments (including tech lesson) | 7 |
| 10 | Sign-off appointment | 2 |
| **Ongoing support** | | |
| 11 | Support Group | 6 |
| 12 | Regular calls / communication | 2 |
| 13 | Relapse | 4 |
| 14 | Additional appointments | 1 |
| 15 | Annual review | 3 |

# Appendix 6 – RRI and project management

## Equity, diversity, and inclusion

We acknowledge that as a predominantly White, able-bodied, and well-educated team, led by a White professional researcher and operating from a White-led and predominantly White university, our experiences might not be the same as those of the participants, let alone of the population of people with SMI and diabetes as a whole. While we undertook this research in good faith and with the best intentions, we are aware that our practices inevitably excluded certain groups, such as those who are not sufficiently fluent in spoken English, those who have not taken part in research previously, for example because they do not engage with conventional health care, and those who are not comfortable participating in group research settings. Similarly, we recognise that our approach to recruiting HCPs from existing networks was not representative and most likely skewed towards reaching those with an interest in the topic area and/or in research in general. Several of the HCPs we approached were unable to participate due to workload pressures.

However, working within these constraints, and within the time and budget available to complete this project, we actively reached out to under-represented groups to try and ensure a varied sample of participants from different Ethnic backgrounds, of different genders, and age groups, and, as far as possible, including differences in health status. While people with SMI and diabetes and their carers are in themselves under-represented and often vulnerable groups, the same pitfalls still apply as when working with the general population regarding equity, diversity, and inclusion. We were led by the NIHR Include framework to address these. The generalisability of the prototype intervention is limited because we ended up working with a predominantly White group of participants. However, the need for cultural adaptation and/or culturally sensitive translation was recognised by participants and the project team throughout the study and is clearly highlighted in this manuscript. This will be addressed in future work which will aim to further test and refine the intervention before potential wider rollout.

## Participant burden and recognition

We acknowledge that participation in a study of this kind is only possible at the expense of other activities. During the sessions, participants were asked to contribute actively and to engage in group discussions with fellow participants and the study team, under the facilitation of HendonCreative. This was clearly described in the participant information sheet and we asked participants to confirm their happiness to continue at the start of each session after sharing the agenda, i.e. allowing the participants to stop or decline further participation once they knew what was planned for each workshop. We also reminded participants regularly of their right to stop participation at any point and encouraged them to take frequent breaks as needed.

In addition, we aimed to minimise participant burden through other means, for example by offering the option to attend sessions online or in person, offering reimbursement for travel expenses for service-users and carers, and providing refreshments at the meeting venue for those attending in person. We cannot guarantee that participants benefitted directly from taking part in this study, but it was our intention to ensure that they have a rewarding and enjoyable experience.

Service-user and carer participants were offered payment for their participation in line with nationally approved payment guidelines, published by the Co-production collective. An hourly rate of £25.00 was offered for each meeting. We are aware that receiving payments can have unintended negative consequences, for example for individuals receiving certain means-tested benefits. We encouraged participants to seek independent expert advice before accepting or declining payment. The responsibility for this was with each participant and we sign-posted reputable sources of information to support this. All participants gratefully accepted the offered payments.

## Study team and governance

JB, the project lead, undertook this piece of work as part of her PhD. In addition to her ample expertise, she has completed and passed mandatory training about data management and research integrity. Ultimate responsibility for the conduct of the work rests with PC as JB’s primary supervisor.

## Study sponsorship

The University of York is the sponsor for this study.

## Research governance and ethics approval

The study was conducted to protect the human rights and dignity of the participant as reflected in the 1996 version of the Helsinki Declaration. In order to protect the study participants, the following provisions were made/upheld: the explicit wishes of the participant were respected including the right to withdraw from the study at any time; the interest of the participant prevailed over those of science and society; provision was made for indemnity by the investigator and sponsor and a contact name for further information or to raise a complaint was provided. The legal basis for this study under the General Data Protection Regulation is ‘task in the public interest’, and participation consent was sought to comply with privacy obligations under English Common Law.

We obtained ethics approval from the Health Sciences Research Governance Committee (HSRGC), Department of Health Sciences, University of York, UK (Reference: HSRGC/2024/616/G) on 9^th^ April 2024.

## Informed consent

Prospective participants were initially contacted informally to find out if they were interested in the study. This happened either face-to-face in the context of meetings relating to other research projects these individuals were engaged with or via email or text message, using existing contact permissions. When we received a positive response at this stage, we shared the invitation letter, participant information sheet, and consent form. Prospective participants were encouraged to complete, sign, and return the consent form before the first workshop. However, logistically this was not always possible due to technical difficulties in accessing the electronic version of the form or due to paper copies being misplaced. In those instances, attendance at the first workshop was taken to indicate consent and participants were supported to complete and sign the consent form during the course of the study or shortly afterwards.

Ongoing consent to participate was confirmed at the start of each session, after the agenda for each workshop was presented and participants knew what to expect. Participants were also reminded regularly of their right to withdraw at any point.

Guided by the Mental Capacity Act 2005, we made sure that the individuals we approached had capacity to consent to participate in this study.

## Participant confidentiality

Participants were informed of their right to confidentiality and its limits, in particular in relation to any disclosures that would place them or other people at the risk of serious harm. All personal data is stored in a password-protected file, using a participant identifier to link participants’ details to any data collected. This information and all data are stored on servers approved by the University of York and are not accessed by anyone outside of the research team.

Participants’ names and addresses are being retained by the University of York for up to 3 years after the study has ended before being destroyed.

At the start of each session, participants were reminded of the rules of engagement such as kindness, respect, and politeness as well as stressing the need for confidentiality. To allow open and honest discussion, there needed to be a mutual understanding that any disclosures made during a session will not be discussed by anyone outside of a session if there is a risk of breaking confidentiality and identifying the participant outside of the research context. Participants were also encouraged to keep themselves safe by only sharing what they felt comfortable to share and by remembering the option to opt out of any part of the workshops they were not comfortable with.

## Data management

In line with the 2018 General Data Protection Regulation and the Research Governance Framework for Health and Social Care Research, anonymised data is being securely archived by the University of York for a minimum of 10 years. Personal data of participants is being stored for up to 3 years after the study has ended for the purpose of disseminating study findings. It is unlikely that this will take longer than 12 months, however, to ensure that participants receive adequate and full information about the study after it has finished, additional time has been allocated.

All information collected during the study is being kept strictly confidential as detailed above. Information is being held securely in paper and/or electronic formats at the University of York. The University of York complies with all aspects of the 2018 General Data Protection Regulation. Operationally this includes obtaining explicit consent from service providers, people with SMI and their family members/friends to record personal details including name, postal and email address, and contact telephone numbers; and appropriate storage, restricted access, and disposal arrangements for people with SMI and family member/friend personal details. All participants have been informed of their rights regarding the personal information stored, including erasure, rectification, and objection.

Data security:

- All data are being stored in accordance with data protection requirements and will be kept either in a locked filing cabinet in a secure office or in the case of electronic data on a secure server with a password protected computer and files.
- Participants’ names and contact details are being stored in a secure place (a locked filing cabinet) and only accessed by JB, or her direct supervisors based at the University of York (PC and NS). Electronic data are password protected and stored on secure servers either hosted or approved by the University of York that are only accessible via password-protected user profiles.

## Participant and researcher safety and wellbeing

JB is recently Good Clinical Practice (GCP) trained and familiar with the application of the underlying principles in practice. Measures were in place to ensure participant safety during the study, including support with travel bookings, frequent breaks, and a distress protocol that would have been followed if needed. Thankfully, the need for this did not arise.

The hybrid sessions were held on University of York premises during normal working hours. Any virtual meetings and the virtual component of hybrid sessions were conducted using Zoom, which is the University of York approved video conferencing software platform.

The study team attended all sessions as a group and there were no instances where members of the team were exposed to any potential risks arising from lone working.

# Appendix 7 – Participant summary report

The participant summary report is available from the Open Science Framework (OSF) and can be accessed using this link : [https://osf.io/4ae3g/files/osfstorage#](https://osf.io/4ae3g/files/osfstorage)

# Appendix 8 – TIDieR checklist

| **TIDieR item** | **Description** |
| --- | --- |
| **1 – Brief name** | To be determined |
| **2 – Why (rationale, theory, or goal)** | The aim of this intervention will be to support people with SMI to use CGM as a means of improving their overall health and wellbeing, including better glucose management. |
| **3 – What (materials)** | Intervention materials will be co-design with service-users, carers, and HCPs in a subsequent study. They are likely to include written materials in accessible language, as well as audio content and visualisations/infographics. Cards that might be used as prompts and reminders around the house (e.g., nutritional advice, portion size recommendation, reminders of healthy glucose range) were suggested by co-design participants. |
| **4 – What (procedures)** | Details of the activities included in the intervention will also be defined at a later date. Co-design participants highlighted the importance of long-term, and trusting therapeutic relationships with their HCPs. They are looking to receive information and guidance, as well as support and encouragement. The need to understand the technical and logistical aspects of CGM was also discussed in the co-design sessions, with participants suggesting specific tech lessons and an ongoing possibility for reasonable adjustments to be made. At the set-up stage, participants suggested that hands on practice with the sensor would be useful to develop the skills to confidently and competently fit and remove the sensor. |
| **5 – Who provided** | There was no consensus among co-design participants about which practitioner or HCP would be best placed to provide CGM support in the context of SMI. Instead, participants consistently highlighted that it was most important that the information be provided by a trusted individual with a good understanding of both diabetes and SMI. |
| **6 – How (mode of delivery)** | Co-design participants strongly advocated for the availability of multiple modes of delivery, including face-to-face contacts in a clinic setting, home visits, telephone appointments and helplines, as well as text-based delivery, for example via email, text messaging, or instant messaging services. A combination of scheduled appointments and opportunities for on demand contact was suggested as potentially beneficial. |
| **7 – Where (location)** | Participants highlighted that a choice of venues and locations might be needed to make the new intervention accessible to as many people as possible. Home and clinic visits, as well as community venues were suggested as potentially useful, depending on the type of contact. |
| **8 – When and how much** | Participants highlighted that flexibility was key in the dosage, frequency, and duration of intervention contacts, suggesting a considerable scope of individual differences in the amount of intervention a person might require or desire. There was consensus that the intervention should be available long-term and not only at the beginning of someone’s CGM user journey. |
| **9 – Tailoring** | Tailoring and personalisation were recognised as key requirements for the success and acceptability of the new intervention. This was discussed predominantly in the context of individual differences, for example in SMI severity. However, the need for cultural and language adaptation was also highlighted, particular in regards to dietary advice. |
| **10 – Modifications** | N/A |
| **11 – How well (intervention fidelity planned)** | N/A |
| **11 – How well (intervention fidelity actual)** | N/A |
